# *PhbZIP2* regulates photosynthesis-related genes in an intertidal macroalgae, *Pyropia haitanensis,* under stress

**DOI:** 10.3389/fmolb.2024.1345585

**Published:** 2024-04-15

**Authors:** Han Zhang, Gaoxiong Zeng, Jiajia Xie, Yichi Zhang, Dehua Ji, Yan Xu, Chaotian Xie, Wenlei Wang

**Affiliations:** ^1^ Fisheries College, Jimei University, Xiamen, China; ^2^ Fujian Engineering Research Center of Aquatic Breeding and Healthy Aquaculture, Xiamen, China; ^3^ Key Laboratory of Healthy Mariculture for the East China Sea, Ministry of Agriculture and Rural Affairs, Xiamen, China; ^4^ Freshwater Fisheries Research Institute of Fujian, Fuzhou, China

**Keywords:** intertidal macroalgae, abiotic stress tolerance, bZIP transcription factor, DAP-seq, heterologous expression

## Abstract

Intertidal macroalgae are important research subjects in stress biology. Basic region-leucine zipper transcription factors (bZIPs) play an important regulatory role in the expression of target genes under abiotic stress. We herein identified a bZIP2 gene *PhbZIP2* to regulate abiotic stress tolerance in *Pyropia haitanensis*, a representative intertidal macroalgal species. Cloning and sequencing of the cDNA characterized a BRLZ structure and an α coiled-coil structure between amino acids and Expression of *PhbZIP2* was detected to upregulate under both high temperature and salt stresses. A DAP-seq analysis revealed the *PhbZIP2*-binding motifs of (T/C)TCCA(C/G) and A (A/G)AAA (G/A), which differed from the conserved motifs in plants. Overexpression of *PhbZIP2* was indicative of a high temperature and salt stress tolerances in transgenic *Chlamydomonas reinhardtii*. It was suggested that *PhbZIP2* was probably involved in regulating expression of the photosynthetic-related genes and the response to the abiotic stresses in *P. haitanensis*, which provide new insights for elucidating efficient adaptation strategies of intertidal macroalgae.

## 1 Introduction

The intertidal zone experiences some of the most drastic environmental changes of any of the world’s ecosystems, but a variety of benthic macroalgae are widely distributed within it. At low tide, these intertidal macroalgae are completely exposed to the air and face multiple abiotic stresses, such as water loss, high temperature, hypersalinity, and high irradiance; at high tide, however, their thalli are completely submerged in seawater, where they quickly emerge from the ‘dormant’ state and return to normal growth and development (Blouin et al., 2011; Bartlett et al., 2017; Wang et al., 2020; Chen et al., 2021). Intertidal macroalgae are therefore an important subject of research in stress biology, and analysis of the adaptive strategies of intertidal macroalgae can provide new ideas for the study of plant adaptive evolution.

*Bangiomorpha pubescens*, possibly extant 1,198–24 million years ago according to fossil evidence, is presumed to have had the strong ability to live in a harsh intertidal habitat during this ancient era (Nicholas, 2000). *Pyropia*/*Porphyra*, a representative genus of intertidal Bangiales, is shaped by stress (Blouin et al., 2011). Many studies have been devoted to the molecular mechanism behind the stress tolerance of this economic seaweed. Under external stress, a potential self-amplifying signaling loop involving Ca^2+^, phosphatidylinositol, and reactive oxygen species (ROS) is generated in this alga (Zheng et al., 2019; Suda and Mikami, 2020; Wang et al., 2020; Guan et al., 2022), and the rapid, unique Ca^2+^ signal is then decoded by calmodulin (CaM) to activate various CaM-dependent kinases (Brawley et al., 2017; Wang et al., 2022). Potential downstream pathways, such as the antioxidant system, are then activated to maintain redox homeostasis (Wang et al., 2018; Wang et al., 2019a; Wang et al., 2020a; Wang et al., 2020b), while heat shock proteins (HSPs), the ubiquitin–proteasome system, and protein processing systems are induced to maintain intracellular protein synthesis, folding, and degradation homeostasis (Wang et al., 2019b; Wen et al., 2020; Niu et al., 2022; Zheng et al., 2022). This ‘sensing-transduction-activation’ model is crucial to the ability of *Pyropia*/*Porphyra* thalli to acclimate and adapt to various stresses. A number of important candidate genes involved in the stress tolerance of *Pyropia/Porphyra* have also been identified by omics technology and molecular biology methods; examples include the superoxide dismutase (SOD) gene *PhSODs* (Ji et al., 2016), *PhHSPs* (Uji et al., 2019; Chang et al., 2021), the RING-type ubiquitin ligase *PhCUL4* (Wang et al., 2021), and the D-group mitogen-activated protein kinase *PyMAPK2* (Kong et al., 2021). The manner in which these candidate genes are activated and regulated is nonetheless still unclear.

When plants respond to abiotic stress, transcription factors can control the expressions of downstream target genes at a specific intensity at a specific time and space to regulate this process. Basic region-leucine zipper transcription factors (bZIPs), which constitute one of the largest, most conserved transcription factor families in plants, play an important role in the regulation of target gene expression in plants under abiotic stress (Jakoby et al., 2002; Droge-Laser et al., 2018). bZIPs contain two domains: a basic region in the N-terminus that recognizes and binds conserved sequences on gene promoters, and a leucine zipper region in the C-terminus that drives activation and repression functions (Hurst, 1995). bZIPs can bind to a downstream gene promoter containing the ACGT core motif in the N-terminus and then regulate the transcriptional expression of downstream stress-related genes to improve plant stress resistance. For example, glutathione activates the promoters of *HSP90.1* via *bZIP10* in *Arabidopsis* (Kumar and Chattopadhyay, 2018). Similarly, overexpression of *ZmbZIP4* can increase the expression of abscisic acid synthesis-related genes, including *NCED*, *ABA1*, *AAO3*, and *LOS5*, and further improve the abiotic stress tolerance of maize (Ma et al., 2018). The role of bZIPs in regulating the response of intertidal macroalgae to abiotic stress, however, is not clear.

The comprehensive identification of transcription factor binding sites helps to characterize regulatory elements and transcription factor functions. Chromatin immunoprecipitation sequencing (ChIP-seq) is currently the leading technology for capturing transcription factor binding site information. This method is highly dependent on gene-specific antibodies, however, and has disadvantages, such as difficult operation and high cost, that limit its popularization and use (Pouya and Manols, 2014). DNA affinity purification sequencing (DAP-seq) is a novel technique for analyzing information on the binding of transcription factors. This approach relies on the construction and expression of transcription-factor proteins *in vitro*, which are then bound to target genome fragments and sequenced. The motif sequences obtained by Dap-seq are compared with the known motif database, and the detected motifs are annotated by the known motifs. DAP-seq is suitable for analyzing species for which transcription factor antibodies cannot be prepared and for investigating less-studied transcription factors. In a comparison of DAP-seq and ChIP-seq data obtained for multiple transcription factors, the overlap rate of DAP-seq and ChIP-seq peaks reached 81%, and the overlap rate with high-scoring motif data was as high as 97% (Brawley et al., 2017). DAP-seq data can therefore be used for peak-calling and motif analysis to obtain potential target genes of transcription factors.

In this study, we identified the key bZIP transcription factor, *PhbZIP2*, from whole genome data of *P. haitanensis* and analyzed its gene structure and expression pattern. After cloning, we obtained information on the binding site and target proteins of *PhbZIP2* by combining ChIP-seq and DAP-seq technology. Finally, we confirmed the function of *PhbZIP2* in abiotic stress response via heterologous expression. Our results can serve as a theoretical foundation for a comprehensive understanding of the abiotic stress tolerance of intertidal macroalgae.

## 2 Materials and methods

### 2.1 Treatment of experimental materials

The experimental strain, *Pyropia haitanensis* Z-61, was selected from the Laboratory of Germplasm Improvements and Applications of *P. haitanensis* at Jimei University (Chen et al., 2019). The control group was cultured at 21°C ± 0.5°C under a light intensity of 50–60 μmol photons m^−2^ s^−1^ and a 12-h light/12-h dark photoperiod. The culture medium, fresh Provasoli’s enrichment solution, was replaced every 2 days. When the cultured algae reached a length of 15 ± 2 cm, undamaged, non-distorted, smooth-planar thalli exhibiting good growth were selected for subsequent experiments. For the high temperature stress treatment, thalli were maintained in a constant temperature incubator at 32°C for 0, 5, 10, 15, 30, 60, 180, or 360 min. According to our previous study, 4 h under 100‰ and 110‰ were selected as hypersaline stress conditions (Chen et al., 2019), and 0‰ and 5‰ were selected as hyposaline stress conditions (Wang et al., 2020a).

The *Chlamydomonas reinhardtii* strain used in this study was selected from the cell wall deletion strain ‘CC-400 CW15 MT+’ cultured in our laboratory. The control group was cultured in a 150-mL conical flask with shaking at 100 rpm min^−1^ and a temperature of 25°C ± 0.5°C under a light intensity of 50 μmol photons m^−2^ s^−1^ and a 14-h light/10-h dark photoperiod. High temperature treatment conditions were the same as those used for *P. haitanensis.* As a hypersaline treatment, the NaCl salinity of the *C. reinhardtii* medium was adjusted to 150 mM, with all other conditions unchanged. Variation in the biomass of treated samples over time (0, 24, 48, and 72 h) was determined by observing sample phenotypes and measuring OD values at 750 nm.

### 2.2 Isolation and purification of total RNA and synthesis of cDNA

Total RNA was extracted from *P. haitanensis* samples using an EZNA plant RNA extraction kit (Omega, USA). The purity and quantity of the extracted RNA were initially estimated from OD_260_ and OD_280_ values measured with a Cary50 UV spectrophotometer (Varian, USA), and RNA integrity and quality subsequently confirmed by 1% agarose gel electrophoresis. The extracted RNA was then reverse transcribed into cDNA using a PrimeScript RT Reagent kit (Takara).

### 2.3 Cloning and expression pattern analysis of *PhbZIP2* from *P. haitanensis*

A bZIP family-related gene, designated as phaG00003535, was screened from the previously generated database of *P. haitanensis* genome and transcriptome (Cao et al., 2020). The identified sequence was cloned by PCR using specific primers. Next, forward and reverse primers for real-time fluorescence quantitative PCR (qRT-PCR) were designed from the cloned sequence, and their quality was assessed using an amplification efficiency of 95%–105% as a criterion. *PhUBC* and *β-tubulin* were used as reference genes with *P. haitanensis* and *C. reinhardtii*, respectively (Supplementary Table S1). A qRT-PCR amplifications were performed using 10× gradient-diluted cDNA as a template to produce standard gene curves on a Step One Plus fluorescence quantitative PCR instrument (ABI, USA). The qRT-PCR cycling protocol for *P. haitanensis* and *C. reinhardtii* was as follows: 95°C for 30 s, followed by 40 cycles of 95°C for 5 s and 58.1°C for 30 s.

### 2.4 Bioinformatics analysis of *PhbZIP2*

The online tool Open Reading Frame Finder (ORF Finder) at NCBI (https://www.ncbi.nlm.nih.gov) was used to analyze *PhbZIP2* nucleotide and amino acid sequences, predict and analyze the open reading frame position, and obtain start and stop codons. Conserved function domains were analyzed with the SMART online tool (http://smart.emblheidelberg.de/), and the primary structure of the protein encoded by the gene was investigated using ExPASy (http://web.expasy.org/protparam/). A phylogenetic tree of bZIP2 protein sequences was constructed by the neighbor-joining method in MEGA6.

### 2.5 Expression of an *PhbZIP2* fusion protein and DNA affinity purification sequencing

A DAP genomic DNA library was prepared and DAP reactions were performed as previously described (Brawley et al., 2017). Briefly, the homology-arm base sequences of a HaloTag expression vector were added to both ends of the start and stop codons of the *PhbZIP2* gene to construct a recombinant vector containing a HaloTag and *PhbZIP2* (Supplementary Table S1). The TnT SP6 high-yield wheat germ protein expression system was used to express the *PhbZIP2*–HaloTag fusion protein, which was purified with HaloTag magnetic beads, washed, and filtered to remove non-specific or unconnected protein. Next, 30–100 ng DNA was added to the fusion protein for co-incubation and further recovery of binding fragments. The DNA library was detected, filtered, and sequenced. Finally, a peak analysis of the read data was performed on the whole *P. haitanensis* genome to identify *PhbZIP2* binding sites. Peaks were scanned with MACS2 analysis software at a threshold of *q < 0.05*, and the information on peak locations was used to screen out peak-related genes. Peak-related genes were annotated with ChIPseeker R package. In addition, MEME (http://meme-suite.org/tools/meme) and DREME (https://meme-suite.org/meme/tools/dreme) were used to detect the sequence motif.

### 2.6 Construction and transformation of the *PhbZIP2* expression vector in *C. reinhardtii*

Base sequences of KpnI and PstI restriction sites were added to the target-gene start and stop codons, respectively. The target gene and the *C. reinhardtii* expression vector pChlamy_3 was then digested with KpnI and Pst I endonucleases, respectively. After double-enzyme digestion, the target gene and the expression vector were ligated overnight at 16°C, and the ligation product was transformed into *E. coli* DH5α receptive cells. Positive monoclonal colonies with ampicillin resistance were selected for PCR verification and expansion culture. The plasmid was extracted with an EndoFree Mini Plasmid kit II to obtain a pChlamy_3 expression vector containing the *PhbZIP2* gene. The circular plasmid vector was linearized by single-enzyme digestion to optimize gene integration into the *C. reinhardtii* genome. The target gene was then transferred into *C. reinhardtii* by the glass bead transformation method (Kindle, 1990), and monoclonal *C. reinhardtii* with hygromycin resistance was screened for propagation and culture for use in subsequent experiments.

### 2.7 Transcriptome analysis

Total RNA from *C. reinhardtii* was sent to Gene Denovo Biotechnology Co., Ltd. (Guangzhou, China) for cDNA synthesis, and construct cDNA library for sequencing. Removing the low quality raw data reads, all remaining high quality clean sequencing reads were mapped onto *C. reinhardtii* genome reference to reconstruct transcripts using Stringtie. After calculating the expression levels of all single genes, the single gene expression levels of two samples were compared. The false discovery rate (FDR) was used to determine the threshold *p*-value for multiple tests. The following criteria were used to identify differentially expressed genes (DEGs): FDR <0.05 and a log_2_|fold-change| ≥ 1. Mapping all differentially expressed genes to each pathway of the KEGG database (https://www.kegg.jp/) and counting the number of genes in each pathway to identify the pathways that are significantly enriched in differentially expressed genes. Tbtools (v1.098769) was used to map and present the analysis of DEGs.

### 2.8 Statistical analysis

Each experimental treatment was performed with three replicates. SPSS 23.0 and Excel were used for statistical analysis of the experimental results. One-way ANOVA was used to compare differences between groups, with *p < 0.05* and *p < 0.01* considered to be significant and extremely significant differences, respectively.

## 3 Results

### 3.1 Cloning and expression pattern analysis of *PhbZIP2*

**FIGURE 1:**
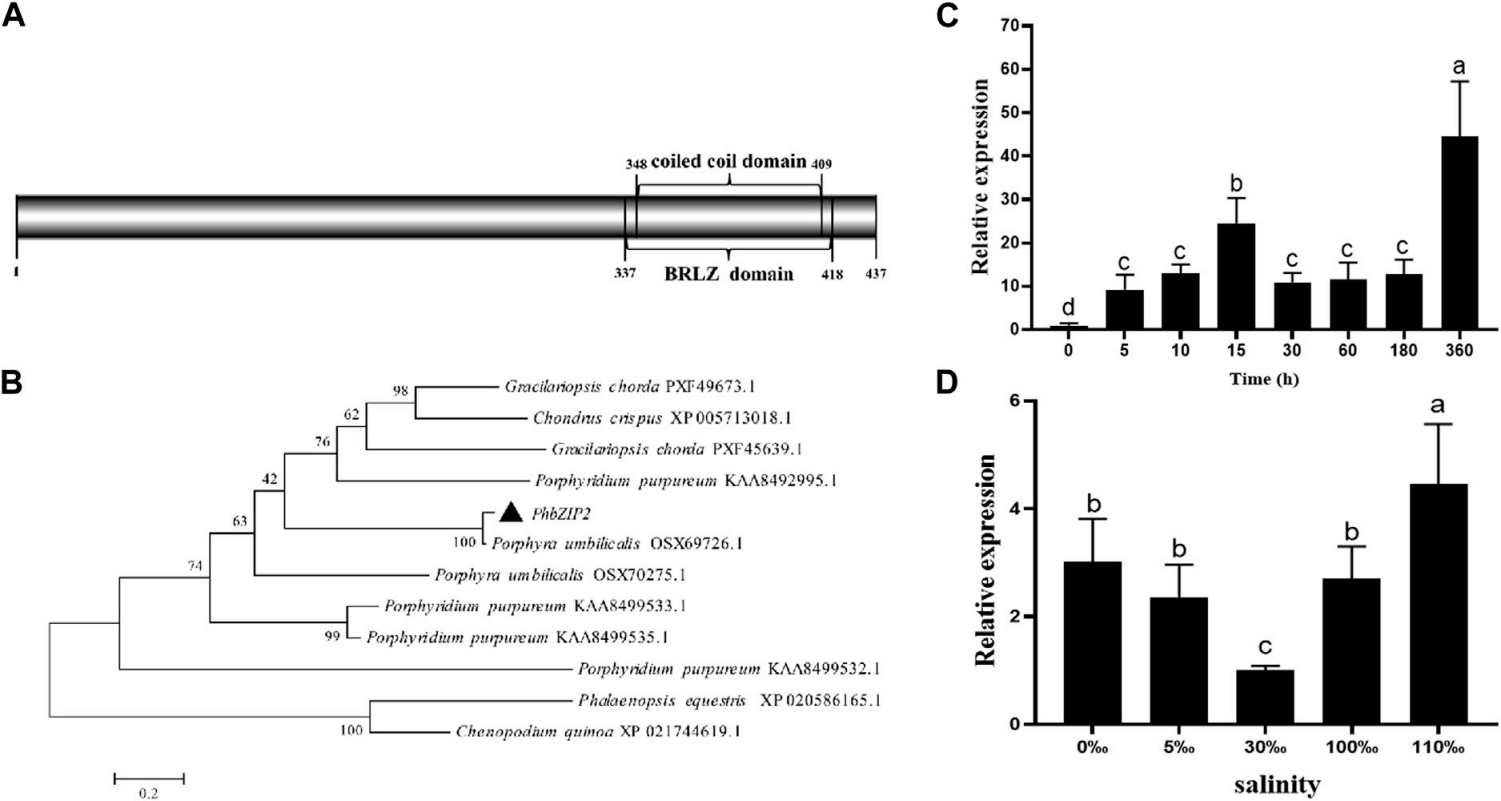
Sequence and expression pattern of *PhbZIP2*. **(A)** Protein domain analysis of *PhbZIP2*; **(B)** Neighbor-joining phylogenetic tree of *PhbZIP2* amino acid sequences, The accession number of each sequence are PXF49673.1 *Gracilariopsis chorda*, XP_005713018.1 *Chondrus crispus*, PXF45639.1 *Gracilariopsis chorda*, KAA8492995.1 *Porphyridium purpureum*, OSX69726.1 *Porphyra umbilicalis*, OSX70275.1 *Porphyra umbilicalis*, KAA8499533.1 *Porphyridium purpureum*, KAA8499535.1 *Porphyridium purpureum*, KAA8499532.1 *Porphyridium purpureum*, XP_020586165.1 *Phalaenopsis equestris*, XP_021744619.1 *Chenopodium quinoa*; **(C)** Changes in transcript levels of *PhbZIP2* under high temperature (32°C) stress; **(D)** Relative transcript levels of *PhbZIP2* following 4 h' different salinity treatments. Salinity means the number of grams of NaCl per liter of water. Data are presented as means ± standard error (*n* = 3). Different superscript letters indicate significance compared to the control (*p < 0.05*).

The cDNA clone of *PhbZIP2* was verified with a PCR-based sequence. The *PhbZIP2* open reading frame, comprised 1,314 bp encoding 437 amino acids. The predicted protein had a molecular weight of 44,761.56 Da and a theoretical isoelectric point of 8.92. Conserved domain analysis revealed that the gene contained one BRLZ (337–418 aa) domain (Figure 1A). Phylogenetic analysis placed *PhbZIP2* in a sister relationship with a *bZIP2* from *Porphyra umbilicalis* (Figure 1B). The detected expression of *PhbZIP2* was significantly induced by high temperature and hypersaline and hyposaline stresses (Figures 1C, D).

### 3.2 Global analysis of PhbZIP2 binding sites in gene functional elements

**FIGURE 2:**
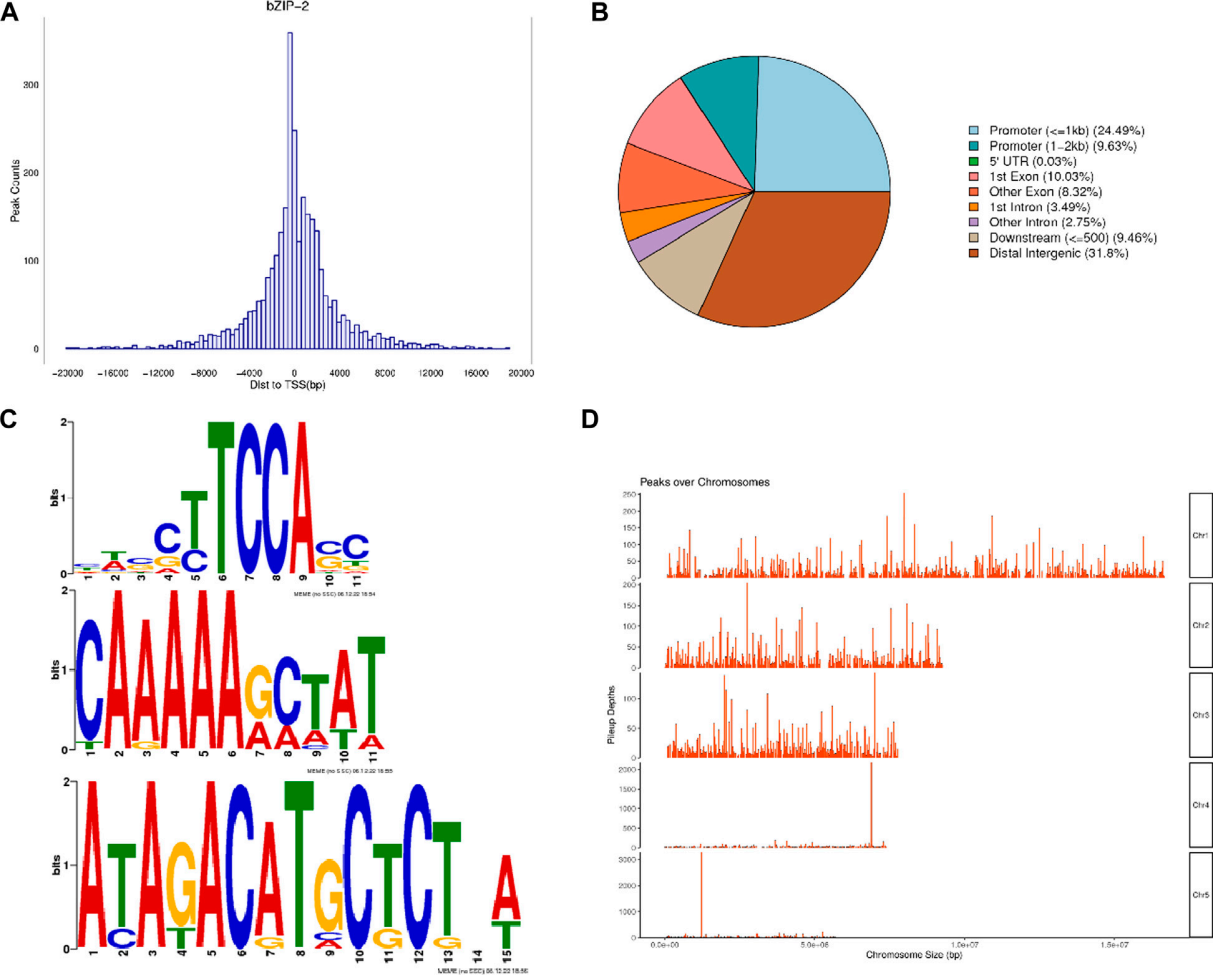
Analysis of *PhbZIP2* binding motifs. **(A)** Number of peaks vs. distance from transcription start sites (TSSs); **(B)** Proportion of peaks in different gene functional elements; **(C)** *PhbZIP2* gene binding-motif sequences; **(D)** Distribution of motif-enriched regions (Peaks) on chromosomes.

The DAP-seq detected a total of 2,981 peaks with a total length of 1,027,663 bp, an average length of 344 bp, a total stacking depth of 47,973, and an average stacking depth of 16 bp. The peak intervals accounted for 2.00% of the genome. The observed distribution of the peaks clustered around 400 bp upstream of transcription start sites (TSSs) (Figure 2A). The overall distribution of peaks was 34.12% in promoters, 0.03% in 5′UTRs, 18.35% in exons, 6.24% in introns, 9.46% in downstream regions, and 31.8% in intergenic regions (Figure 2B). *PhbZIP2* can bind to conserved motifs, such as (T/C)TCCA(C/G), A (A/G)AAA (G/A) and A (T/C)A (G/T)AC (A/G)T (Figure 2C). The motif-enriched regions detected by the DAP-seq were mainly located on Chromosome1, two and 3 (Figure 2D). The significantly enriched KEGG pathways (*p* < 0.01) of the target genes of *PhbZIP2* were involved in Carbon fixation in photosynthetic organisms, Photosynthesis, and Photosynthesis-antenna proteins (Table 1).

**TABLE 1:** The significantly enriched KEGG pathways of the target genes of *PhbZIP2*.

Pathway	*p*-Value	Q value	Pathway ID
Carbon fixation in photosynthetic organisms	0.002157	0.201536	ko00710
Photosynthesis	0.003732	0.201536	ko00195
Photosynthesis-antenna proteins	0.008574	0.283314	ko00196
Pentose phosphate pathway	0.010493	0.283314	ko00030
Mismatch repair	0.013158	0.284210	ko03430
Carbon metabolism	0.020280	0.321429	ko01200
Amino sugar and nucleotide sugar metabolism	0.020833	0.321429	ko00520
Autophagy - other	0.024660	0.332915	ko04136
Carotenoid biosynthesis	0.030152	0.338561	ko00906
Nucleotide excision repair	0.031348	0.338561	ko03420

### 3.3 Identification of stress tolerance in PhbZIP2-expressed C. reinhardtii

**FIGURE 3:**
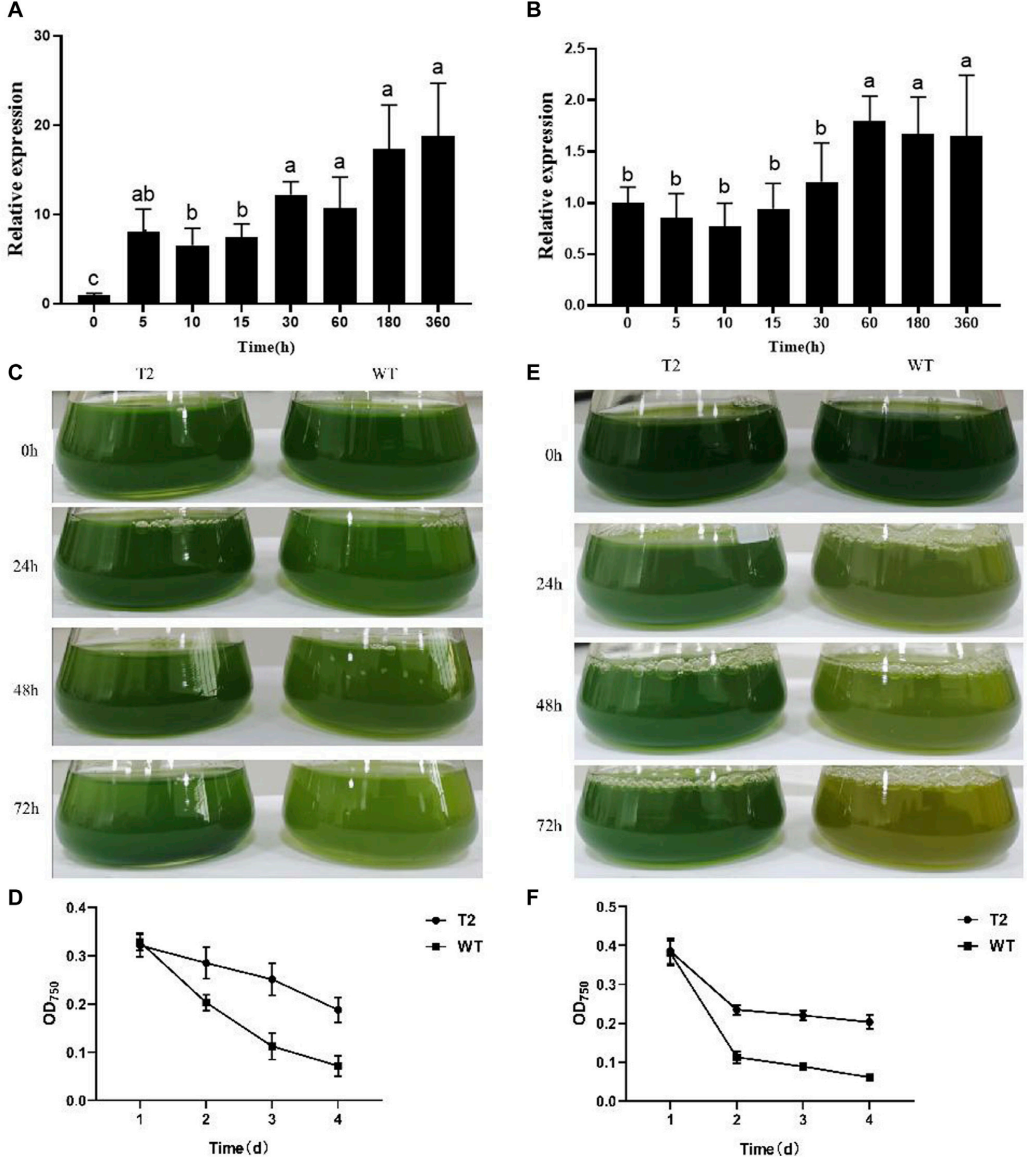
Stress tolerance of wild type (WT) and *PhbZIP2*-expressed transgenic (T2) *C. reinhardtii*. **(A,B)**: Changes in transcript levels of *PhbZIP2* in transformed *C. reinhardtii* at 32°C **(A)** and hypersaline **(B)** stresses; **(C,D)**: Changes in the phenotype **(C)** and biomass **(D)** of transgenic and wild type *C. reinhardtii* under high temperature (32°C) stress. **(E,F)**: Changes in the phenotype **(E)** and biomass **(F)** of transgenic and wild type *C. reinhardtii* under hypersalinity stress. Data are presented as means ± standard error (*n* = 3). In **(A,B)**, different superscript letters indicate significant differences between groups (*p < 0.05*). In d and f, asterisk indicate significant differences between samples at the same time point (*, *p < 0.05*; **, *p < 0.01*).

As confirmed by agarose gel electrophoresis, *PhbZIP2* target fragments were amplified from *C. reinhardtii*, whereas no PCR products were detected from wild type *C. reinhardtii* (Supplementary Figure S1). In transgenic individuals maintained at 32°C, the transcription level of *PhbZIP2* was significantly increased at 5 min and gradually increased as the high temperature stress was prolonged up to 360 min (Figure 3A). In transgenic *C. reinhardtii* subjected to hypersaline stress, the expression level of *PhbZIP2* was relatively stable for the first 30 min. After 60 min of treatment, however, the expression level was significantly increased and then remained at a high level (Figure 3B). It was observed that the survival rate of transgenic *C. reinhardtii* after 48 h of high temperature treatment was always higher than that of the wild type, and the biomass difference between the two lines became increasingly significant with lengthening treatment duration (Figures 3C, D). After treatment with 150 mM NaCl for 72 h, most of the wild type individuals died, whereas transgenic *C. reinhardtii* was still alive (Figures 3E, F).

### 3.4 KEGG enrichment of differentially expressed stress--related genes in transgenic *C. reinhardtii* with *PhbZIP2*

**FIGURE 4:**
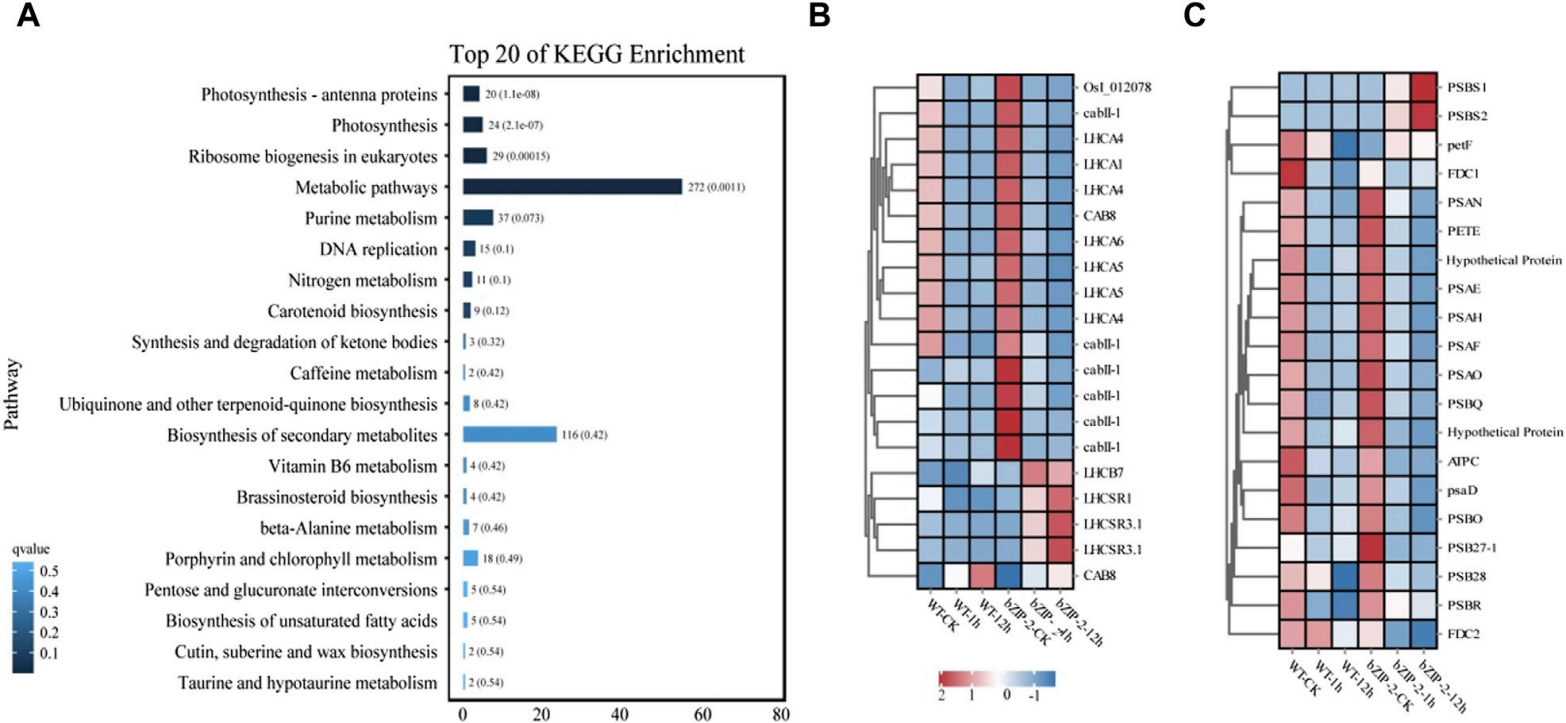
Comparative transcriptome analysis of the wild type (WT) strain and the transgenic strain of *Chlamydomonas reinhardtii* with *PhbZIP2* under salt stress. **(A)** KEGG pathway enrichment analysis of genes under 12 h of salt stress treatment. **(B,C)** represents the effect of stress on the expression of genes annotated by photosynthesis–antenna proteins and photosynthesis, respectively. WT-CK, the sample of WT strain under normal condition; WT-1h, the sample of WT strain treated with salt stress for 1 h; WT-12h, the sample of WT strain treated with salt stress for 12 h; bZIP-2-CK, the sample of the transgenic *C. reinhardtii* strain with *PhbZIP2* under normal condition; bZIP-2-1h, the sample of the transgenic *C. reinhardtii* strain with *PhbZIP2* treated with salt stress for 1 h; bZIP-2-12h, the sample of the transgenic *C. reinhardtii* strain with *PhbZIP2* treated with salt stress for 12 h; Abbreviated gene names are listed in Supplementary Table S2.

Analysis of the KEGG pathway enrichment displayed that the DEGs employed in response to 12 h salt stress were mainly involved in photosynthesis, photosynthesis, photosynthesis-antenna proteins and Ribosome biogenesis in eukaryotes (Figure 4A). The subsequent analysis identified most genes related to photosynthesis-antenna protein had downregulated expression trends in response to salt stress. In the wild type, 17 of 20 and 19 of 20 genes involved in photosynthesis and photosynthesis-antenna proteins, respectively, were downregulated under salt stress treatment. Compared with the wild type, the expression of photosynthesis-related genes (PSBS1, PSBS2, petF) and photosynthesis antenna protein-related genes (LHCB7, LHCSR1, LHCSR3.1, CAB8) were upregulated in transgenic lines under salt stress (Figures 4B, C).

## 4 Discussion

In this study, we found that *PhbZIP2* of *P. haitanensis* contains a BRLZ domain as well as an α coiled-coil structure between amino acids 337 and 418 (Figure 1A), which is typical of bZIP family members (Jakoby et al., 2002). According to the structural differences of bZIP transcription factors, they can be divided into 10 subfamilies. Different family members are widely involved in a variety of biological processes, among which H class is mainly involved in the regulation of photosynthesis (Blouin et al., 2011). The results of phylogenetic tree showed that *PhbZIP2* was clustered with *AtbZIP56* (H class) and other transcription factors, indicating that *PhbZIP2* had high homology with *Arabidopsis thaliana,* and the protein structure type and function had certain similarity. *PhbZIP2* expression was significantly upregulated in response to high temperature almost immediately (starting at 5 min of stress). This outcome is similar to results obtained in studies of wheat and rice, where significant increases in *TabZIP60* and *OsbZIP23* expressions were observed during early stages of high temperature and drought stresses, respectively (Geng et al., 2018; Zong et al., 2020). The upregulation of *PhbZIP2* may therefore serve as a rapid response to abiotic stress to activate downstream stress-resistance genes. The expression of *PhbZIP2* obviously decreased after 15 min of high temperature treatment, however, and then significantly increased at 360 min (Figure 1C). Wang et al. (2018) have reported that the response of *P. haitanensis* to high temperature stress can be divided into two stages. First, in the early stage of stress, the thallus had a stress response, which reduced photosynthesis to reduce the production of reactive oxygen species and unnecessary energy consumption. Afterwards, recombination was performed at the transcriptional level to further activate stress-resistant pathways such as photosynthesis, energy metabolism, and antioxidant systems to resist long-term high temperature stress. At 360 min of heat stress, in the present study, the upregulation of bZIP transcription factor may help activate stress-related genes such as photosynthesis to meet the energy and material needs of the thallus. (Wang et al., 2018). In addition, *PhbZIP2* expression in transgenic *C. reinhardtii* was significantly increased at 5 min and 60 min of high temperature and hypersaline stress treatments, respectively (Figures 3A,B). The survival rate of *PhbZIP2*-expressing transgenic *C. reinhardtii* was always higher than that of the wild type under continuous high temperature or hypersaline stress (Figures 3C–F). These results indicate that *PhbZIP2* is a key transcription factor regulating the transcriptional expression of downstream stress resistance-related genes in *P. haitanensis*.

Most *PhbZIP2* peaks obtained from DAP-seq were concentrated near the TSS, with 34.12% and 31.8% distributed in gene promoters and intergenic regions, respectively (Figures 2A, B). This result suggests that *PhbZIP2* regulates the transcription of downstream genes by binding to their promoters or to intergenic regions. Plant bZIP proteins preferentially bind palindromic or pseudo-palindromic *cis*-acting elements containing an ACGT core sequence, such as ABREs (CCACGTGG) and A-box (TACGTA), C-box (GACGTC), and G-box (CACGTG) elements (Dröge-Laser et al., 2018). These proteins can also bind to some motifs of non-palindromic structures (Sornaraj et al., 2016). For example, rice RF2a and tomato VSF-1 bZIP transcription factors can bind to the non-palindromic promoter sequences CCCACCTACCA and TCA​CCA​ACC​GTT​GGA​TGT​GG, respectively (Yin et al., 1997; Ringli and Keller, 1998). Our analysis revealed that (T/C)TCCA(C/G) and A (A/G)AAA (G/A) motif sequences are abundant in *PhbZIP2* (Figure 2C). We hypothesize that the motifs bound by *PhbZIP2* differ from those with conserved binding regions in plants.

The significantly enriched (*p* < 0.01) KEGG pathways that were common to both the target genes of *PhbZIP2* were Carbon fixation in photosynthetic organisms, Photosynthesis, and Photosynthesis-antenna proteins (Table 1). Photosynthesis of *Pyropia/Porphyra* is found to be highly sensitive to abiotic stresses (Wang et al., 2018; Terada et al., 2021; Shao et al., 2022). Increasing in light-harvesting efficiency and light-energy use, and suppressing accumulation of ROS are crucial for *P. haitanensis* responses to abiotic stress (Ji et al., 2023). bZIP transcription factor BLZ8 expression increased the photosynthetic linear electron transfer rate, decreasing the excitation pressure of the photosynthetic electron transport chain, and in turn inhibiting the accumulation of ROS in *C. reinhardtii* under oxidative stress (Choi et al., 2022). The *Arabidopsis* transgenics overexpressing a *bZIP* gene from *Triticum aestivum*, *TabZIP*, also exhibited higher photosynthetic efficiency and increased tolerance to salt stress (Agarwal et al., 2019). Similarly, the DEGs between the transgenics *C. reinhardtii* overexpressing *PhbZIP2* and wild type under salt stress were also enriched (*p* < 0.01) by Photosynthesis and Photosynthesis-antenna proteins (Figure 4). Overall, the major of these DEGs showed downregulation in wild type and transgenics lines under salt stress, which was consistent with the results of reduction of biomass (Figure 3), indicating salt treatment impaired the photosynthesis of algae. But *PhbZIP2* increased the expression levels of most DEGs involved in photosynthesis and photosynthesis-antenna proteins under normal condition. Additionally, in comparison with wild type, one PsbR genes, two PsbS genes, three LHCSR genes, and one LHCB genes exhibited obvious upregulation in transgenics lines under salt stress. This was beneficial for transgenics lines to protect against photo-oxidative damage to photosynthesis organelles during prolonged salt stress. Therefore, photosynthesis-related genes may be important target genes for transcription factor *PhbZIP2* regulation in response to abiotic stress.

## 5 Conclusion

In this study, the bZIP family transcription factor *PhbZIP2* in *P. haitanensis* was cloned and characterized for the first time. Transcription of *PhbZIP2* were rapidly accumulated during the high temperature and saline stresses. DAP-seq and heterologous expression analyses suggested that *PhbZIP2* was involved in the response of *P. haitanensis* to abiotic stress by regulating photosynthesis, which shed light on the stress resistance mechanisms of intertidal seaweed and provide important information for breeding new varieties with stronger stress resistance.

## Data Availability

The datasets presented in this study can be found in online repositories. The names of the repository/repositories and accession number(s) can be found below: https://www.ncbi.nlm.nih.gov/, PRJNA839642.
