# Supplementary material for: PhbZIP2 regulates photosynthesis-related genes in an intertidal macroalgae, Pyropia haitanensis, under stress
Source: Front Mol Biosci. 2024 Apr 15;11:1345585. doi: 10.3389/fmolb.2024.1345585 (PMC11056619; doi:10.3389/fmolb.2024.1345585)
Supplement: Supplementary file 2 [file Table1.DOCX]

Table S1 Primers and sequences used in this experiment

| Species | Primer name | Use | Sequence |
| --- | --- | --- | --- |
| *Pyropia haitanensis* | *PhbZIP2F* | PCR amplification | CTCCCATCCGTCGTTATCTC |
|  | *PhbZIP2R* |  | CATCCGTTTTCTTTTCGTTG |
|  | *PhbZIP2QF* | qRT-PCR | GGACTTTTCGCCCCTTACC |
|  | *PhbZIP2QR* |  | ACGCAAACGCACTCGCTAC |
| *Chlamydomonas reinhardtii* | *SODQF* | qRT-PCR | CAAGGTCCTTGAGCTCGG |
|  | *SODQR* |  | AGCACCACCAGACCTACGTGA |
|  | *CATQF* |  | TCCGCGGGGTCCATGGTCTG |
|  | *CATQR* |  | CGCCCGTCATCGTGCGGTTC |
|  | *HSP70AQF* |  | AGCACTGATGCTGTGTTTCG |
|  | *HSP70AQR* |  | CATGTTGGCAAAATCAATCG |
|  | *HSP70BQF* |  | AGTCCACCCAAGTGCCTTACC |
|  | *HSP70BQR* |  | GCTGGGAGTCATTGAAGTAGGC |
|  | *HSP90AQF* |  | GCTCAAGAAGATGGGCTACG |
|  | *HSP90AQR* |  | GTGGCTGACCATGACCTTCT |
|  | *HSP90CQF* |  | AGAAGAAGAAGGCGGAGGAG |
|  | *HSP90CQR* |  | GCTGTCTAGTAGGCGGTTGG |
| *Pyropia haitanensis* | *PhUBCF* | Reference genes | TCACAACGAGGATTTACCACC |
|  | *PhUBCR* |  | GAGGAGCACCTTGGAAACG |
| *Chlamydomonas reinhardtii* | *TubulinQF* |  | CTCGCTTCGCTTTGACGGTG |
|  | *TubulinQR* |  | CGTGGTACGCCTTCTCGGC |
| *Halo Tag-PhbZIP2 expression vector* | *PhbZIP2 DapF* | *PhbZIP2* amplification (Homologous arm base) | ACTTTCAGAGCGATAACGCG  ATGAGTTACATGCCGTACAAC |
|  | *PhbZIP2 DapR* |  | TACCGAGCCCGAATTCGTTT  TCATGCGCCCGCAATTGCCCG |
